# Ultrasound Confirmation of the Multiple Loci Hypothesis of the Myofascial Trigger Point and the Diagnostic Importance of Specificity in the Elicitation of the Local Twitch Response

**DOI:** 10.3390/diagnostics12020321

**Published:** 2022-01-27

**Authors:** Andrew Ball, Thomas Perreault, César Fernández-de-las-Peñas, Michael Agnone, Jordan Spennato

**Affiliations:** 1 Atrium Health, Carolinas Rehabilitation, Charlotte, NC 28211, USA; Michael.Agnone@atriumhealth.org (M.A.); Jordan.Spennato@atriumhealth.org (J.S.); 2 Myopain Seminars, 4405 East-West Highway, Suite 401, Bethesda, MD 20814, USA; Thomas.Perreault@WDHospital.org; 3 NxtGen Institute, 2138 Scenic Highway, Snellville, GA 30078, USA; 4 Wentworth-Douglass Hospital Rehab Services at Dover, 789 Central Avenue, Dover, NH 03820, USA; 5 Department of Physical Therapy, Occupational Therapy, Rehabilitation and Physical Medicine, Universidad Rey Juan Carlos, 28922 Madrid, Spain

**Keywords:** ultrasound, trigger point, dry needling, injection, case series

## Abstract

The literature has hypothesized that a trigger point (TrP) area consists of a hyperperfused contracture knot with smaller hypoperfused TrPs within the contracture knot. By contrast, the only published ultrasound image of a TrP has it labeled hypoechoic (i.e., hyperperfused) with no commentary regarding smaller speckles of hypoperfusion within. Furthermore, the lack of clarity in objective definition of the terms associated with the TrP (namely, the palpable “contracture knot” and smaller nonpalpable “trigger point”) has led to unnecessary communication difficulties between and among clinicians and researchers. In this case series of three muscles across two patients, by using high-definition musculoskeletal ultrasound imaging technology, we present what we believe to be the first reliable capture of palpable hypoechoic (e.g., hypoperfused) contracture knots (previously mislabeled as a hypoechoic TrP), and a visual support of the multiple loci hypothesis first proposed by Hong and Simons—the first reliable confirmation of the hyperechoic (i.e., hypoperfused) TrP within. Initially proposed by a histological study and supported by microdialysis study, this case series lends further support for the multiple loci hypothesis through visual confirmation of palpable hypoechoic contracture knots, with smaller hypoechoic TrPs “speckles” within.

## 1. Introduction

Characterized as a motor and/or sensory disorder, or myofascial trigger point (TrP) that refers pain [1], myofascial pain syndrome is estimated to affect from 30% to 85% of the population [2]. The integrated hypothesis of TrP formation is the most widely accepted model describing TrP formation in the skeletal muscle. It involves a cascade of events including the leakage of acetylcholine into the synaptic cleft and the release of calcium into the sarcoplasmic reticulum, resulting in a nonvolitional muscle contraction at the motor endplate [3]. Considering the theoretically small size of the TrP, some authors have questioned if TrPs can be palpated with accuracy [4,5].

Clinicians use manual palpation to identify TrPs, with “spot tenderness” and “referred pain” among the most common features for TrP identification [6,7]. However, recent research indicates the reliability of manual palpation is questionable and has poor reproducibility for eliciting TrP-related symptoms [8]. For example, needling of TrPs was shown to produce referred pain and local twitch responses (LTR) when prior manual palpation failed to do so [9,10]. It has been traditionally accepted that maximal target specificity of the dry needling technique is advisable when an LTR is the procedural goal [11]. Nevertheless, it has been recently observed that, although the LTR elicited during dry needling can be clinically relevant, clinical outcomes on pain and function are not directly related just to the fact of eliciting LTR [12,13]. Accordingly, the therapeutic value of the LTR is a current hotly debated topic in the literature [14].

A dual-action effect of dry needling integrating updated pain neuroscience mechanisms is currently hypothesized [15]: a first local biomechanical effect mitigated by needle manipulation of collagen fibers, and a second neurological/sensory effect of more theoretical importance in centrally sensitized patients [15]. The literature to date has not delineated patients with and without central sensitization in a study of the clinical value of the LTR [13]. Nevertheless, clinicians performing TrP injection (TrP-I) or TrP dry needling (TrP-DN) hold to empirical evidence that for optimal clinical results, specificity wherein the needle contacts the precise target is paramount. However, the specificity of targeting the TrP area during dry needling procedure has been recently questioned when applying this intervention in individuals with stroke [16].

Two microdialysis studies revealed ischemia and hypoxia within TrP areas with surrounding hyperperfusion [17,18]. Proper understanding of ultrasound imaging (US) suggests that, in general, hyperperfused structures appear as hypoechoic (dark grey) and hypoperfused structures as hyperechoic (light grey) [19]. Previous US studies had instead identified and labeled TrPs as hypoechoic (dark grey) [20,21]. A hypoechoic TrP image suggests either anisotropy (image error) or, in direct contrast to widely accepted micro-dialysis literature [17,18], that the TrP is a hyperperfusion area.

Over the years, the terms “contracture knot” and “TrP” have been used interchangeably [22,23]. This lack of clarity in objective definition of these two terms has led to unnecessary communication difficulties among clinicians and researchers. Hong proposed that the sensorimotor abnormalities of TrPs are related to multiple sensitized afferent nerves [24] and motor endplates [25] contained within the “TrP region,” or what clinicians define as a palpable “contracture knot”. Meng et al. identified an increased number of afferent nerve fibers in the “TrP region” compared to normal muscle tissue and concluded that proliferation of afferent nerve fibers plays a significant role in TrP pathophysiology [26]. Recent studies on animal models support the idea that contracture knots are collections of smaller TrPs located at the neuromuscular junctions [27], but this phenomenon has not been visualized to date with US. An old previous attempt at US of TrPs failed to reliably identify contracture knots due to limitations in the imaging technology at the time of the study [28]. In the following years, advancements in the technology of the ultrasound probe and computerized imaging processing have resulted in dramatic advancements in image quality [29,30]. In fact, a recent study used US imaging for assessing the intensity of the LTR during the application of dry needling [31]; however, this study failed to visualize the TrP area itself under US.

We present here two patients in whom we were able to capture the contracture knots, by using high-definition US technology, representing the first visual confirmation of Hong’s multiple loci hypothesis [24]. Accordingly, we describe three muscles across two single patients, where we visualized that the palpable and hyperperfused contracture knot is actually a collection of much smaller nonpalpable hypoperfused TrPs at motor endplates residing within the palpable contracture knot.

## 2. Case Report #1

The first case was a 6′2″, 185 lbs., 53-year-old male subject (previously evaluated by both primary care physician and doctor of physical therapy to rule out other causes of symptoms other than myofascial TrPs) with dual complaints of intermittent right plantar foot and right shoulder pain previously successfully treated by TrP dry needling of the gastrocnemius and deltoid, respectively.

**Figure 1:**
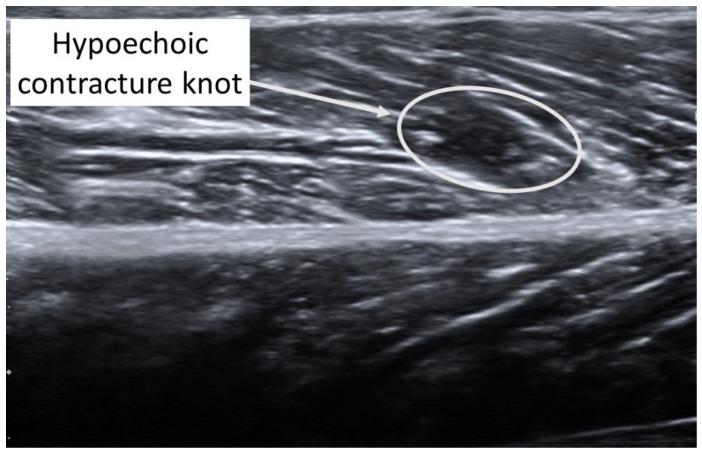
Ultrasound imaging assessment of the right gastrocnemius muscle showing the palpable contracture knot as a hypoechoic (hyperperfused) area.

**Figure 2:**
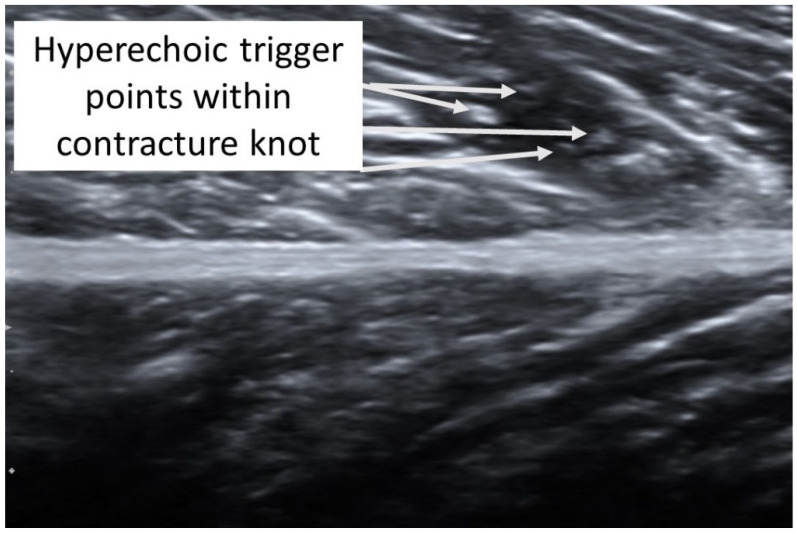
Detailed ultrasound imaging assessment of the right gastrocnemius muscle showing a collection of small hyperechoic (hypoperfused) “TrP speckles” within the contracture knot.

A taut band with a palpable nodule within the medial gastrocnemius was identified as responsible for his right plantar heel pain symptoms [32]. As had been the case with previous bouts of plantar pain, the subject reported, upon palpation, a pain referral symptomatology into the plantar aspect of his foot as his pain symptoms (pain recognition). The nodule was subsequently imaged in long-axis with a Siemens Acuson S200 Ultrasound system (Siemens, Munich, Germany) with an 18L6 16 Hz high-definition linear probe. A large hypoechoic contracture knot (109 mm × 47 mm) was identified (Figure 1) with smaller hyperechoic “speckles” (Figure 2) within the hypoechoic contracture knot.

**Figure 3:**
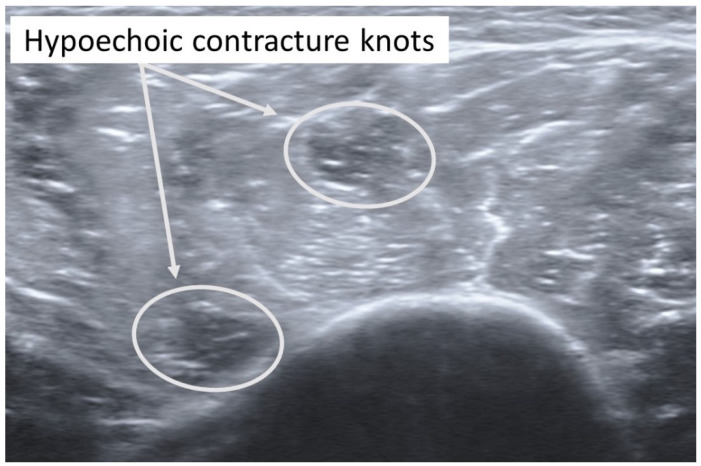
Ultrasound imaging assessment of the right deltoid muscle showing two palpable contracture knots as hypoechoic (hyperperfused) areas.

**Figure 4:**
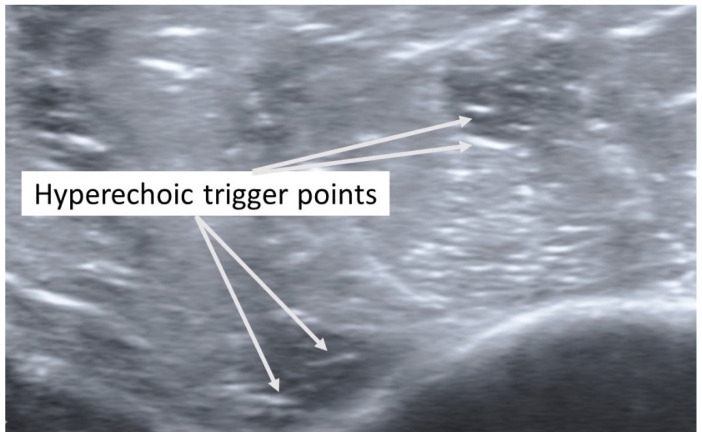
Detailed ultrasound imaging assessment of the right deltoid muscle showing a collection of small hyperechoic (hypoperfused) “TrP speckles” within each of the contracture knots.

In addition, taut bands with palpable nodules responsible for his right shoulder pain were also identified in the right anterior and middle deltoid muscles. As had been the case with previous bouts of shoulder complaints, the subject reported, upon palpation, a referred pain symptom spreading down the anterior and middle aspect of his upper extremity. The spots were subsequently imaged in short-axis with a Siemens Acuson S200 Ultrasound system with an 18L6 16 Hz high-definition linear probe. Two large hypoechoic contracture knots (Figure 3) (both ~75 mm × ~75 mm) were identified with smaller hyperechoic “speckles” (Figure 4) within each hypoechoic contracture knot.

## 3. Case Report #2

**Figure 5:**
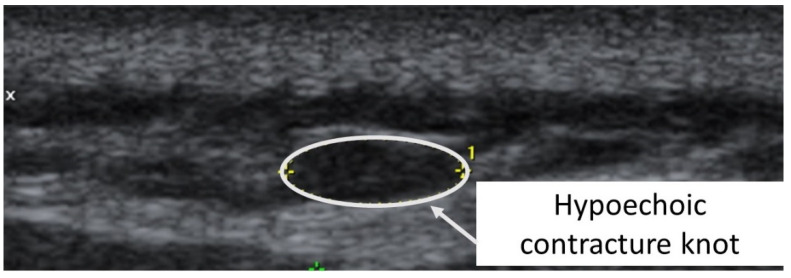
Ultrasound imaging assessment of the left upper trapezius muscle showing a palpable contracture knot as a hypoechoic (hyperperfused) area.

**Figure 6:**
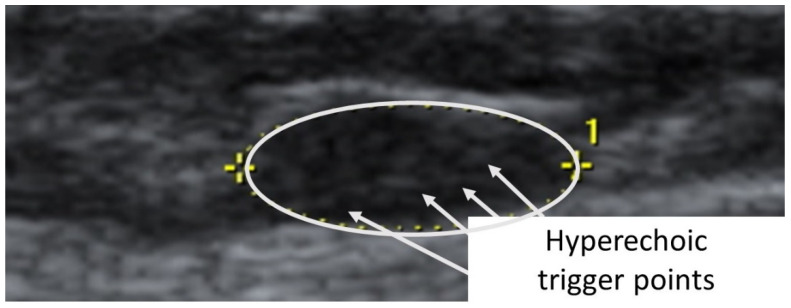
Detailed ultrasound imaging assessment of the left upper trapezius muscle showing a collection of small hyperechoic (hypoperfused) “TrP speckles” within the contracture knot.

The second case was a 5′6”, 130 lbs., 26-year-old female subject (previously evaluated by both primary care physician and doctor of physical therapy to rule out other causes of symptoms other than TrPs) with left temporal headaches previously successfully treated by TrP-DN of the left upper trapezius. A taut band with palpable nodule within the left upper trapezius muscle was identified. As had been the case with previous headaches, the subject reported, upon palpation, pain referral symptoms spreading up the neck, around the ear, and into the temple [32]. The nodule was subsequently imaged in long-axis with a General Electric Voluson i Ultrasound System with a 12L-SC 13 MHz linear probe. A large hypoechoic contracture knot (61 mm × 22 mm) was identified (Figure 5) with smaller hyperechoic “speckles” (Figure 6) within the hypoechoic contracture knot (approximately 1 mm × 1 mm).

## 4. Discussion

Considering that the pathogenesis of TrPs consists of dysfunctional motor endplates and they are ischemic areas in nature, previous studies may have mislabeled contracture knots as TrPs [19]. In each case presented, a hypoechoic/hyperperfused area of approximately 1 cm × 1 cm was visualized in the vicinity of the palpable nodule, with small hyperechoic/nonpalpable speckles of approximately 1 mm × 1 mm within each. Consistent with the multiple loci hypothesis presented by Hong and Simons [25], we suggest that the relatively large hypoechoic structures visualized as the TrP area in both subjects examined represent hyperperfused contracture knots with smaller, not previously distinguished, ischemic and hyperechoic TrPs within each.

**Figure 7:**
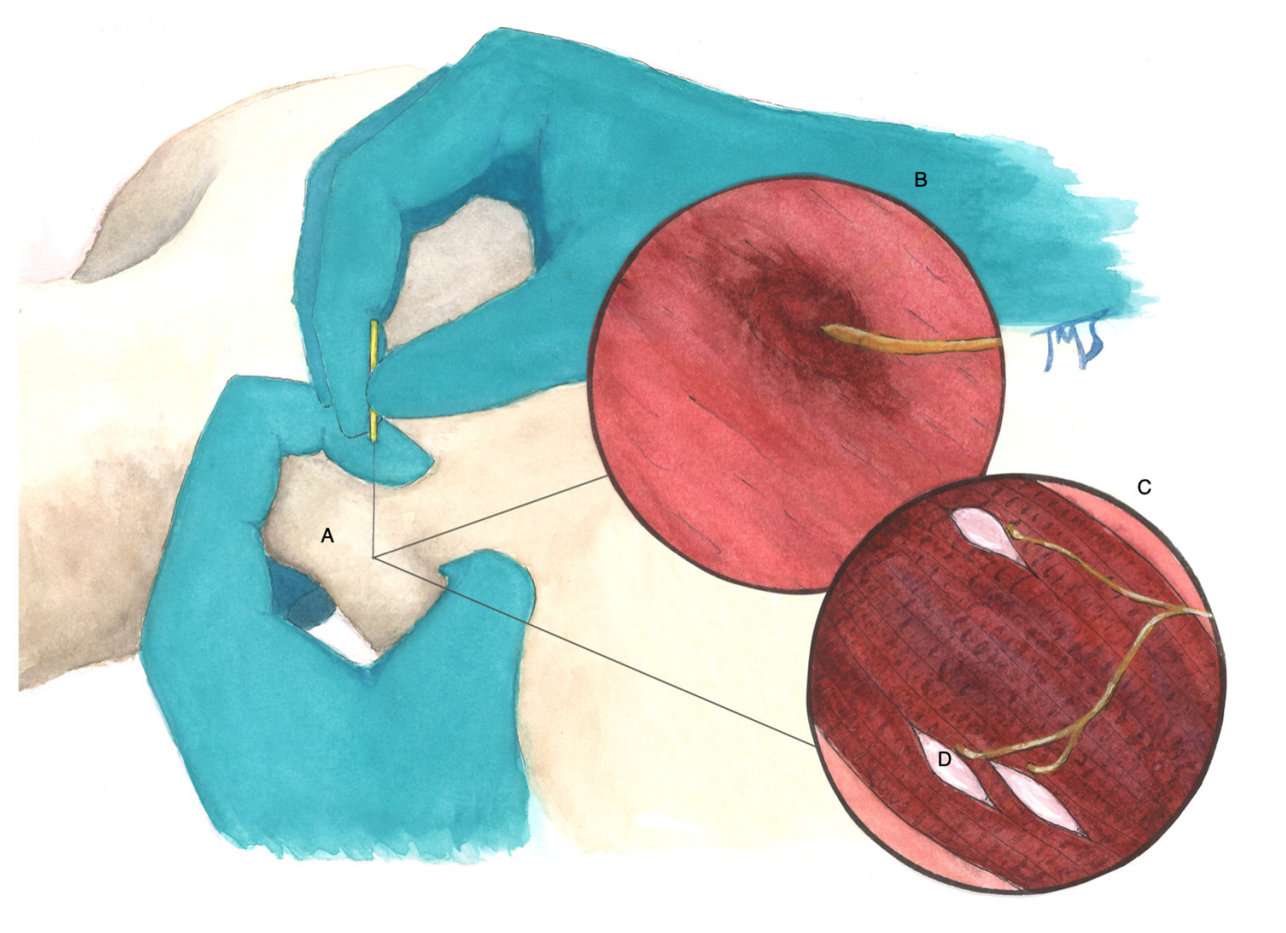
Illustration of the multiple loci hypothesis. (**A**) Palpable, tender nodule located within a taut band of skeletal muscle; (**B**) hyperperfused, hypoechoic contracture knot and nearby motor nerve; (**C**) multiple hypoperfused and hyperechoic trigger points located within a contracture knot; (**D**) region of multiple active loci (motor end plates) and sensitive loci (sensitized afferent nerve endings).

The visual confirmation of contracture knots in the muscle being hyperperfused challenges the idea that the therapeutic benefit of dry needling is related to a “wash out effect” that somehow results in reperfusion of what we now suggest is an already hyperperfused region [20,21]. We propose a new hypothesis suggesting that when performing TrP-DN, an initial therapeutic effect may occur due to decreasing (not increasing) perfusion of hyperperfused contracture knot relative to the surrounding skeletal muscle. In addition, elicitation of a more specific perfusion-homeostatic effect likely occurs upon needle stimulation of the smaller hyperechoic (e.g., hypoperfused) TrPs within the contracture knot. Consistent with the multiple loci hypothesis (Figure 7), it is conceivable that the needle tip also pricks the afferent nerve endings that are sensitized within and around the hypoperfused TrPs to elicit analgesic effects [33]. It is possible that the mechanisms underlying TrPs are much more complex than previously realized.

### 4.1. Limitations

Although hypoechoic images are generally considered hyperperfused and hyperechoic images are generally considered hypoperfused [19], the possibility of image artifact (anisotropy) in our patients cannot be ruled out. For example, acoustic shadow behind calcification, lymph nodes, and some select pathological conditions may present as hypoechoic areas. Furthermore, small, curved, superficially located musculoskeletal structures (such as tendon and joint structures) and deep muscular structures requiring the use of a low-frequency, low-definition curvilinear probe are particularly susceptible to anisotropy [34]. We believe that in the cases presented, the probability of anisotropy is quite low secondary to the use of a high-frequency linear probe to examine superficial structures. Anisotropy would represent theoretical incongruence with both current understanding of the biochemical milieu of the contracture knot and TrP [17,18], and the multiple loci hypothesis [24].

### 4.2. Clinical Relevance

Currently, the therapeutic value of eliciting an LTR during dry needling is a matter of professional opinion [14]. However, the evaluative ability for “needle palpation” to elicit an LTR as an objective sign of TrP presence is less debatable [11,13,33]. We propose, based on current data, that the LTR is not necessarily elicited by needle stimulation of the contracture knot, but it is elicited by needle stimulation of smaller TrPs within the contracture knot—as previously theorized [11,13,33,35,36] and for the first time visualized and identified—in the cases presented. 

Additionally, the visual confirmation of contracture knots in a muscle being hyperperfused challenges the idea of dry needling deriving part of its therapeutic benefit via a “wash out effect” that somehow results in reperfusion [37] of what we now suggest is an already hyperperfused region. Since this is just a case series including two cases, visual confirmation of the multiple loci hypothesis is a necessary consideration toward accurate structure identification and labeling for professional communication, research, education, and procedural target specificity of dry needling interventions.

## 5. Conclusions

In clinical procedures such as TrP-DN where a LTR is preferable obtained, optimization of target specificity is key. Initially supported by a histological study [17,38], this case series lends further support for the multiple loci hypothesis through visual confirmation of palpable contracture knots with smaller TrPs within.

## Data Availability

Not applicable.
